# Supplementary material for: Genetic Divergence and Population Structure of Xanthomonas albilineans Strains Infecting Saccharum spp. Hybrid and Saccharum officinarum
Source: Plants (Basel). 2023 May 9;12(10):1937. doi: 10.3390/plants12101937 (PMC10222335; doi:10.3390/plants12101937)
Supplement: Supplementary file 1 [file plants-12-01937-s001.zip › plants-2371006-supplementary.pdf]

Table S1: Sizes and sequence identities (%) of five housekeeping genes of 39 *X. albilineans* strains from China.

Gene	Nucleotide		Amino Acids	
	Length (nt)	Identity (%)	Length (aa)	Identity (%)
ABC	608	99.6 – 100	202	99.5 – 100
*gyr*B	904	99.3 – 100	300	99.3 – 100
*rpo*D	1298	98.4 – 100	432	97.9 – 100
*atp*D	868	97.0 – 100	289	97.9 – 100
*gln*A	1095	97.6 – 100	364	97.5 – 100

Table S2: Primers used for the amplification of five house-keeping genes of 39 *X. albilineans* strains from China.

Target Genes [a]	Primers [b]	Sequence (5′→3′)	NCBI Accession Numbers [c]
ABC	XAF1	CCTGGTGATGACGCTGGGTT	MT776083-MT776094, MT776096-MT776106, MT776108-MT776122, MH709206
	XAR1	CGATCAGCGATGCACGCAGT	
*atp*D	atpD-F2	GGGCAAGATCGTTCAGAT	MT776144-MT776148, MT776150-MT776155, ON112168-ON112195
	atpD-R2	GTTCTTGGTGGAGGTGAT	
*gln*A	glnA-F2	GGTTAAGGACAACAAGATCG	MT776160-MT776164, MT776166-MT776171, ON112107-ON112125, ON112131-ON112139
	glnA-R2	GCGGCRAAGGTCAGGTAG	
*gyr*B	XgyrB1F	ACGAGTACAACCCGGACAA	MT776038-MT776049, MT776051-MT776061, MT776063-MT776077, MH709220
	XgyrB1R2	CCCATCAAGGTGCTGAAAAT	
*rpo*D	rpoD-F1	ATGGCCAACGAACGTCCTGC	MT776128-MT776132, MT776134-MT776139, ON112140-ON112167
	rpoDR3	AACTTGTAGCCGCGACGGTATTC	

[a] *ABC*, ABC transporter protein; *rpo*D, encoding the β subunit of the bacterial RNA polymerase; *gln*A, encoding a citrate synthase; *gyr*B, β subunit of the DNA gyrase; *atp*D, β subunit of ATP synthase.

[b] The primer pair XAF1/XAR1 and other four primer pairs were referred to Wang et al. (2010) and Ntambo et al. (2019), respectively.

[c] The sequences of *ABC* gene were from this study, while the sequences of other four genes were retrieved from the observation by Zhao et al. (2022).

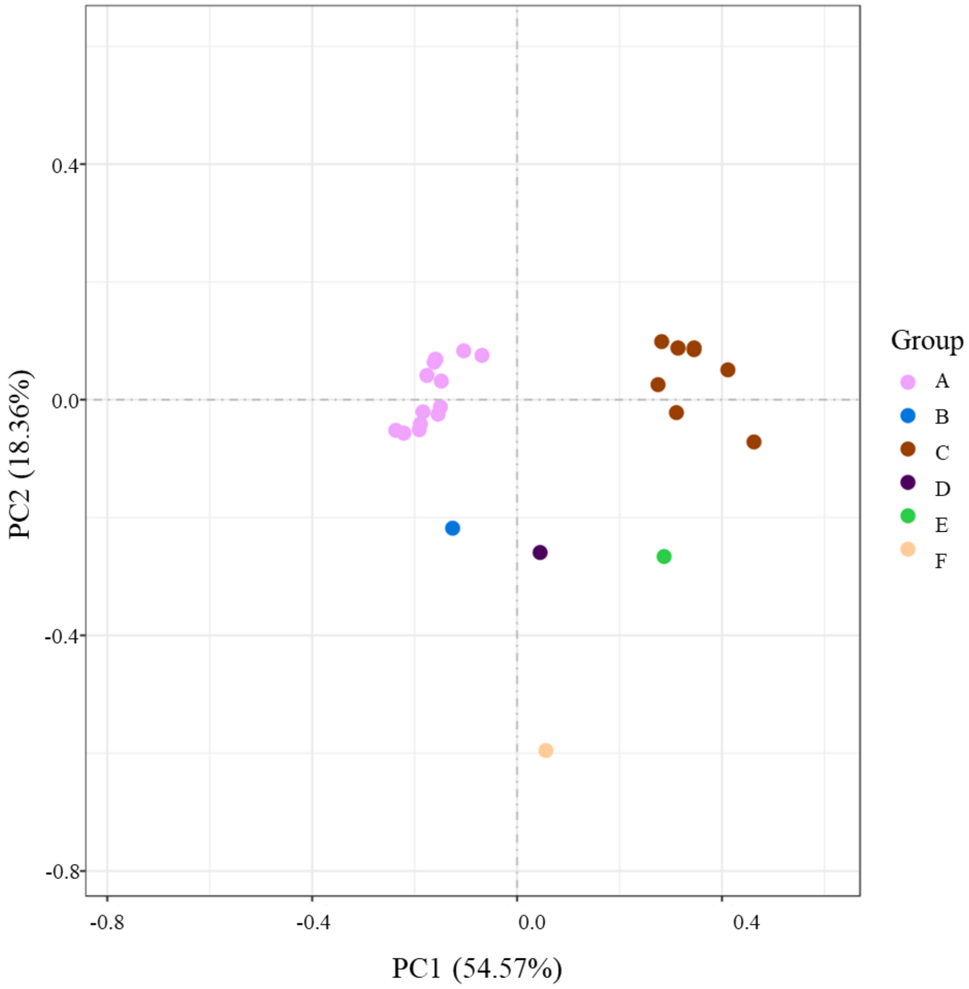

Figure S1: Principal coordinates analysis of 39 *X. albilineans* strains of based on rep-PCR fingerprints.
